# Dysgraphia as a Mild Expression of Dystonia in Children with Absence Epilepsy

**DOI:** 10.1371/journal.pone.0130883

**Published:** 2015-07-01

**Authors:** Renzo Guerrini, Federico Melani, Claudia Brancati, Anna Rita Ferrari, Paola Brovedani, Annibale Biggeri, Laura Grisotto, Simona Pellacani

**Affiliations:** 1 Pediatric Neurology and Neurogenetics Unit and Laboratories, Neuroscience Department, A. Meyer Children’s Hospital, University of Florence, 50139, Florence, Italy; 2 IRCCS Stella Maris Foundation, 56128, Pisa, Italy; 3 Department of Statistics, Informatics, Applications “G. Parenti”, University of Florence, Florence, Italy; University of Pennsylvania Perelman School of Medicine, UNITED STATES

## Abstract

**Background:**

Absence epilepsy (AE) is etiologically heterogeneous and has at times been associated with idiopathic dystonia.

**Objectives:**

Based on the clinical observation that children with AE often exhibit, interictally, a disorder resembling writer’s cramp but fully definable as dysgraphia, we tested the hypothesis that in this particular population dysgraphia would represent a subtle expression of dystonia.

**Methods:**

We ascertained the prevalence of dysgraphia in 82 children with AE (mean age 9.7) and average intelligence and compared them with 89 age-, gender- and class-matched healthy children (mean age 10.57) using tests for handwriting fluency and quality, based on which we divided patients and controls into four subgroups: AE/dysgraphia, AE without dysgraphia, controls with dysgraphia and healthy controls. We compared the blink reflex recovery cycle in children belonging to all four subgroups.

**Results:**

We identified dysgraphia in 17/82 children with AE and in 7/89 controls (20.7 vs 7.8%; P = 0.016) with the former having a 3.4-times higher risk of dysgraphia regardless of age and gender (odd ratio: 3.49; 95% CI 1.2, 8.8%). The AE/dysgraphia subgroup performed worse than controls with dysgraphia in one test of handwriting fluency (P = 0.037) and in most trials testing handwriting quality (P< 0.02). In children with AE/dysgraphia the blink reflex showed no suppression at short interstimulus intervals, with a difference for each value emerging when comparing the study group with the three remaining subgroups (P<0.001).

**Conclusions:**

In children with AE, dysgraphia is highly prevalent and has a homogeneous, distinctive pathophysiological substrate consistent with idiopathic dystonia.

## Introduction

Absence epilepsies are etiologically heterogeneous. Genetic causes include mutations in different genes such as *SLC2A1* [1], *BK* [2], *GABRG2* [3], *CACNB4* [4], *CACNA1A* [5], and *CLCN2* [6]. Causal heterogeneity, in turn, underlies variable pathophysiological backgrounds, as suggested by the association of absence epilepsy with paroxysmal dyskinesia, cognitive impairment, or other seizure types in later life. Co-occurrence of absence epilepsy and paroxysmal dyskinesia, in particular [7], suggests a common dysfunction of cortico-subcortical networks.

We noticed that a substantial number of children in our cohort of typical absence epilepsy of childhood (CAE) exhibited a distinct handwriting disorder resembling writer’s cramp but fully definable as dysgraphia, a disorder of written expression featuring writing skills substantially below those expected considering age and intelligence [8]. Dysgraphia implies seriously poor legibility or low writing speed, or both, interfering with the child’s educational achievements and with daily activities requiring these abilities [9]. The exact prevalence of dysgraphia is unknown but handwriting problems among school age children have been estimated to vary between 5 and 33% [10–11].

Based on the clinical characteristics of patients in our cohort and previous reports about possible associations between absence epilepsy and dyskinesia [7], we tested the hypothesis that these children’s dysgraphia would reflect an underlying dystonic disorder.

## Methods

### Participants

We ascertained the prevalence of dysgraphia in a cohort of children with absence epilepsy, which included 131 patients consecutively evaluated between 2005 and 2011 at the Neurological Unit of the Meyer Children’s Hospital, Florence, and the Research Institute Stella Maris Foundation, Pisa. We selected patients who met the following inclusion criteria:

1. Normal neuro-developmental milestones, age-appropriate neurological examination, and neuroimaging; epilepsy onset before the age of 10 years; ictal EEG with bilateral, synchronous, symmetrical spike-waves at around 3 Hz; brief (4 to 20 seconds, exceptionally longer) and frequent (>10 per day) absence seizures with abrupt impairment of consciousness; typical absence seizures as the only seizure type, or associated with occasional generalized tonic-clonic seizures or preceded by febrile seizures.
2. Age at the time of the study ranging between 8–13 years, an age range in which a diagnosis of dysgraphia can be made.
  Normal cognitive function as defined by school performance and clinical assessment. We performed formal testing in patients exhibiting mild learning problems which made us suspicious of borderline cognitive skills.
3. Absence of serious linguistic and/or learning disorders. To rule out concomitant disorders in linguistic and phonological abilities, we administered fluency tasks [12]. To exclude reading disorders (dyslexia) we evaluated reading speed and accuracy (expressed in number of errors) by asking patients to read ‘word’ and ‘non word’ sequences [13]. To exclude dysorthographia we administered an uninterrupted dictation of 10 lines [14].
4. Absence of specific motor disorders on clinical examination. We tested the organization of fine movements and visuo-spatial integration according to subtests BVN 5–11 [15] and BVN 12–18 [16] and Visual Motor Integration [17].

**Fig 1:**
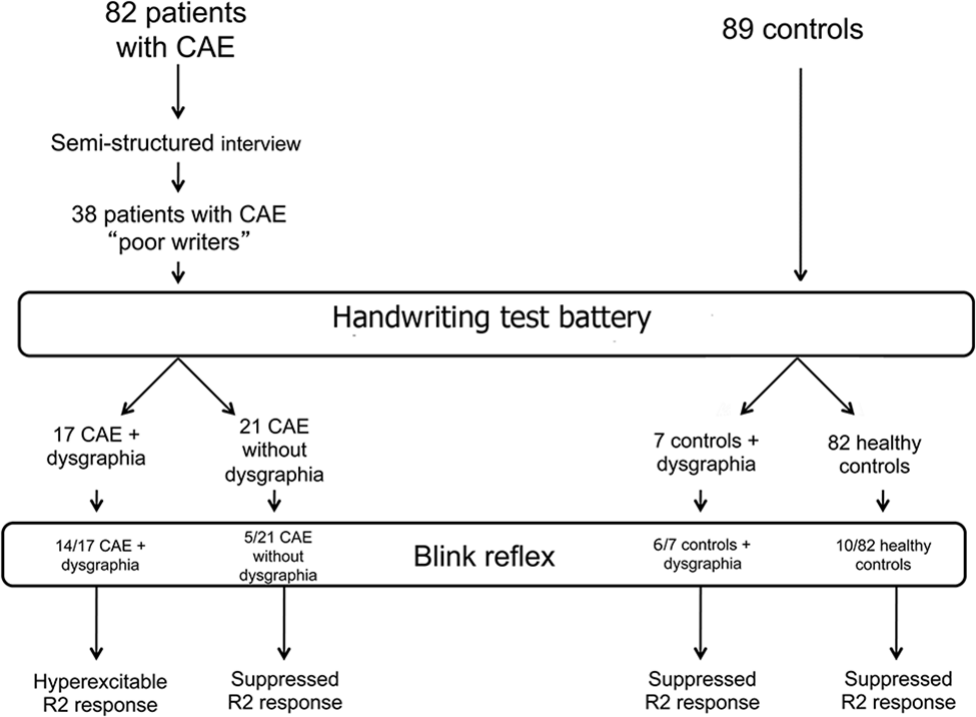
Diagram of the study.

Using the above criteria, we selected 82 patients (mean age 9.7; SD 1.78; 53.7% females) (Fig 1). We labelled as “poor writers” 38 /82 patients (mean age 9.67; SD 1.73; 57.9% females) based on assessment of two pediatric neurologists who examined recent and remote, school and homework handwriting samples. Whenever seriously poor legibility or exceedingly slow writing speed, or both, were apparent, a child neuropsychologist formally tested children using a handwriting test battery [18–19]. We subsequently divided “poor writers” with absence epilepsy into two subgroups: children with absence epilepsy/dysgraphia and children with absence epilepsy without dysgraphia.

We compared our cohort with a control group of 89 age-,gender- and class-matched children (mean age 10.57; SD 1.44, 52.8% males). In order to be eligible, controls, likewise the study group, had to have: 1, normal neurodevelopmental milestones, age appropriate neurological examination; 2, age at the time of the study ranging between 8–13 years; 3, normal cognitive level as defined by school performance and clinical assessment; 4, absence of serious linguistic and/or learning disorders; 5, absence of obvious motor disorders upon clinical examination. Controls were enrolled from primary and secondary public schools in Tuscany. We tested the 89 children in the control group using the same handwriting test battery as in the study group and, based on results, divided them into controls with dysgraphia and controls without dysgraphia (healthy controls).

This study was approved by the ethical committees of the Meyer Children’s Hospital and the Research Institute Stella Maris Foundation. All clinical investigations were conducted according to the principles expressed in the Declaration of Helsinki. Written informed consent was obtained from the parents and caretakers on behalf of all patients and control subjects enrolled in the study.

### Handwriting test battery

To assess the exact prevalence of dysgraphia among the 38 ‘poor writers’ with absence epilepsy and in the control group, we tested both groups with a handwriting test battery administered in two separate test sessions assessing handwriting speed and legibility/quality [18–19]. Children were considered to have dysgraphia if their handwriting performances were characterized by low writing speed or poor legibility, or both.

Twelve patients with absence epilepsy/dysgraphia were re-tested 5 years apart using the same handwriting test battery.

#### Handwriting fluency

We administered two tests: the “UNO” (one-uno uno uno) test and the “LE” (lelelele) test [18]. The child was asked to write the word “UNO” (ONE) for a minute, then to write an alternated sequence of cursive letters (“l” and “e”) for a minute with a continuous movement. We calculated the total number of readable allographs in each test. Two scores were obtained and children were rated to have performed poorly if they failed at least one of the two tests (cut-off: z score ≤2 SD).

#### Handwriting quality

To evaluate graph-motor and posture handwriting disorders, we administered a standardized test (DGM- P test) [19].In brief, children were asked to copy the Italian sentence “*l’elefante vide benissimo quel topo che rubava qualche pezzo di formaggio*” (the elephant clearly saw the mouse stealing some pieces of cheese), in which each letter of the Italian alphabet is repeated at least twice. Each child was asked to carry out the exercise in two ways, during their “best” and “faster” handwriting condition.

The analysis was based on 12 indexes (S1 File), providing information about 4 areas of interest: visuo-spatial component, motor efficiency, motor pattern, learning. Children were considered to have dysgraphia when their level in the DGM-P test was inadequate, in both the ‘best’ and ‘faster’ condition.

### Neurophysiology

As children with absence epilepsy/dysgraphia manifested abnormal force and posturing in holding the pen, reminiscent of writer’s cramp [20–21], we investigated them with polymyographic recordings at rest and during handwriting and using the blink reflex recovery cycle.

#### Polymyographic study

We performed polymyographic recordings in 10 children with absence epilepsy/dysgraphia. A pair of surface electrodes was placed bilaterally over the deltoid muscles and over the trapezium, triceps, biceps and forearm flexors and extensors on the dominant hand side. Electromyographic (EMG) activity was recorded at rest and during handwriting. In five of the 10 children surface EMG was recorded again 5 after years.

#### Blink reflex recovery cycle

We studied 14 children with absence epilepsy/dysgraphia, using surface recording electrodes placed bilaterally over the inferior orbicularis oculi muscle; reference electrodes were placed laterally on the lateral canthus. Electrical stimuli were applied to the supraorbital nerve in proximity of the supraorbital foramen. Acquisition parameters were: low pass filter: 2 kHz; high pass filter: 20 Hz; sweep time: 100 milliseconds. The usual blink response was represented by an ipsilateral disynaptic response (R1) and a bilateral multisynaptic response (ipsilateral R2 and contralateral R2c). Recordings were performed using a five-channel computerized EMG System–MYOQUICK 1400ME Matrix Line—Micromed s.r.l. (Mogliano Veneto, TV, Italy). The R2 recovery cycle was obtained by delivering a series of paired electrical stimuli: the first stimulus evoked the conditioning response and the second the test response. A total of 10 trains of stimuli were delivered with a progressive increase in the interstimulus interval (ISI) between the two-paired stimuli. The ISI ranged from 100 to 1000 milliseconds (ms), with a 100 ms increase for each train. Each response was rectified and the average value of five trials for each ISI was computed. The excitability of the recovery cycle was defined by comparing the test with the conditioning response (R2 t/c ratio). The R2 t/c ratio for each ISI value was obtained by calculating the area of the test response as a percentage of the conditioning response. The mean values of the R2 t/c ratio were plotted against each ISI to construct a blink reflex recovery curve.

To exclude any influence of valproate (VPA) treatment on the excitability of the R2 recovery curve, we studied 5 children with absence epilepsy without dysgraphia treated with VPA, hypothesizing a normal blink reflex recovery curve. To evaluate if the neurophysiological findings were specific for dysgraphia in the context of absence epilepsy and not for dysgraphia itself, we also studied 6 control children exhibiting dysgraphia as an isolated manifestation. Normative data were obtained from 10 age-matched healthy controls.

The blink reflex recovery cycle was re-evaluated in 5 children with absence epilepsy/dysgraphia 5 years after the first study (follow-up subgroup), using the same neurophysiological procedure

### Genetic testing

Since the association of absence epilepsy and dystonia has been reported in some patients with *SLC2A1* (or *GLUT1*) mutations [22], we tested this gene in 8 children with absence epilepsy/dysgraphia. We studied all exons and intron-exon boundaries of *SLC2A1* by direct sequencing of genomic DNA. We also performed multiplex ligation-dependent probe amplification analysis using the SALSA MLPA Kit P138 *SLC2A1* (MRC- Holland, Amsterdam, the Netherlands) on genomic DNA of 2 patients who were mutation-negative to sequencing. Because of negative results on these patients, we opted not to extend genetic testing to the whole study group.

### Data analysis

We used the STATA 11 (T Stat s.r.l.) for statistical analysis and descriptive statistics (means, standard deviation) to describe the participants’ main variables. To compare the proportions of individuals with dysgraphia in the absence epilepsy group versus that in the control group, we computed the equality of proportions. To compare writing features (fluency and handwriting quality) differences, regardless of age, gender and class between the absence epilepsy group and the control group, we used linear and logistic regressions. To compare writing fluency differences among the four subgroups (absence epilepsy with and without dysgraphia, controls with and without dysgraphia—Fig 1), we used an independent sample Student’s *t*-test. We used the F test for the homogeneity of variances and the *t*-test for unequal variance to evaluate differences between the four areas of interest of the DGM-P test within four subgroups. Handwriting performances of the subgroup of children with absence epilepsy/dysgraphia who were re-tested 5 years later were compared using a paired *t*-test. The blink recovery curves obtained for the four subgroups were compared using regression for repeated measures. The blink recovery curves of the subgroup of children with absence epilepsy/dysgraphia who were re-tested after 5 years were compared using ANOVA for repeated measures. When appropriate, confidence intervals (CI) were calculated. Level of significance was set at 5% two-sided.

## Results

### Prevalence of dysgraphia in cohorts

**Fig 2:**
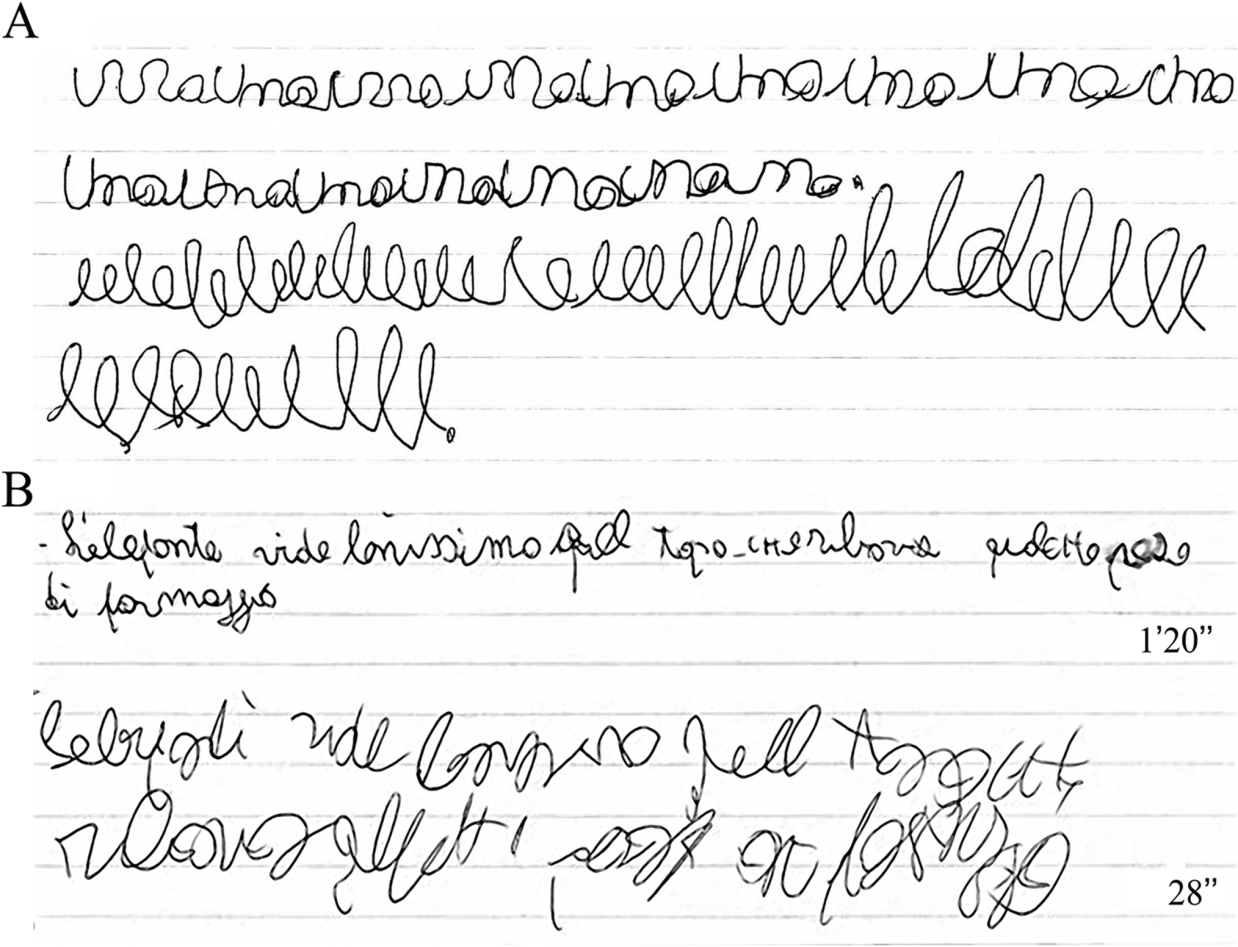
Handwriting performance. (A) Patient 12. Example of handwriting during “uno “(above) and “le” (below) test. The child performed slowly. (B) Patient 9. Example of handwriting performance during ‘best’ (above) and ‘faster’ (below) conditions. We considered this patient’s performance as inadequate.

Using the study battery illustrated in the ‘Methods’ section, we identified dysgraphia in 17/82 children with absence epilepsy and in 7/89 controls (20.7 vs 7.8%; a difference of 12.9%, 95% CI 2.5, 23.3%; P = 0.016) (an example of handwriting of children with absence epilepsy/dysgraphia is illustrated in Fig 2). Children with absence epilepsy had a 3.4-times higher risk of having dysgraphia than controls, regardless of age and gender (OR: 3.49; 95% CI 1.2, 8.8%). The average age of children with absence epilepsy/dysgraphia at the time of the study was 9.6 years (SD 1.6; 52.9% males). The average age at onset of absence seizures was 6.28 years (SD 2.5). Thirteen patients were seizure-free on medication at the time of the study while the remaining four still manifested sporadic absence seizures. Cognitive level was average in all patients and IQ testing was performed in 10/17 patients. The five patients re-tested after 5 years remained seizure free during the follow-up period. Clinical data are summarized in Table 1.

**Table 1:** Summary of clinical features in 17 children with absence epilepsy/dysgraphia.

Patient/sex	Age at absence onset (yrs)	Other seizures (type/age)	Dysgraphia first noticed onset (yrs)	Treatment at time of testing (yrs)	Age at time of testing (yrs)	Absence seizures at time of testing	Cognitive level
1/M*	9.5	No	8	VPA+ETS	11.2	Sz free	Average
2/M*	2.6	GTCS/7yrs	7	VPA	8.9	Sz free	Average
3/F*	3.6	No	7	VPA	8.2	Sz free	IQ:88
4/M	2.6	No	7	ETS	8	Sz free	Average
5/M*	5	No	8	VPA	11.6	Sz free	IQ:102
6/M*	4.4	FS/12m	7	VPA+LTG	13	Sporadic	IQ:83
7/F* °	7	No	6.6	VPA	8.5	Sz free	IQ:88
8/F*	9	No	7	VPA+ETS	10	Sporadic	Average
9/F* °	9.9	No	7	VPA	10.6	Sz free	IQ:82
10/F* °	7.9	FS/24m	8	VPA	8.1	Sporadic	Average
11/F* °	5.6	No	7	VPA+ETS	7.9	Sz free	IQ:84
12/M	5	No	7	No treatment	12.3	Sz free	Average
13/M	4.4	FS/17m	6	VPA+ETS+LTG	8.2	Sz free	Average
14/F*	7.7	FS/36m	7	VPA+LTG	8.1	Sz free	IQ:116
15/F*	4	FS/8m	5.6	VPA+ETS	8.5	Sporadic	IQ:96
16/M* °	9	No	7	VPA	10.3	Sz free	IQ:106
17/M*	9.6	No	9	VPA	9.8	Sz free	IQ:84

F: females; M: males; yrs: years; m: months; FS: febrile seizures; GTCS: generalized tonic clonic seizures; sz: seizure; ETS: ethosuccimide; LTG: lamotrigine; VPA: valproic acid; IQ: intelligence quotient

*: patients studied with blink reflex

°: patients re-tested 5 years after the first test with blink reflex

### Handwriting fluency and quality

When comparing performances of children with absence epilepsy with those of controls in the “UNO” and “LE” tests, using regression analysis, we observed a reduction of 0.79 (95% CI -1.14,-0.45) and 1.32 (95% CI -1.72,-0.94) of the z-score (P<0.001), corresponding to an overall worse performance in children with absence epilepsy, with and without dysgraphia, regardless of age, gender and class.

**Fig 3:**
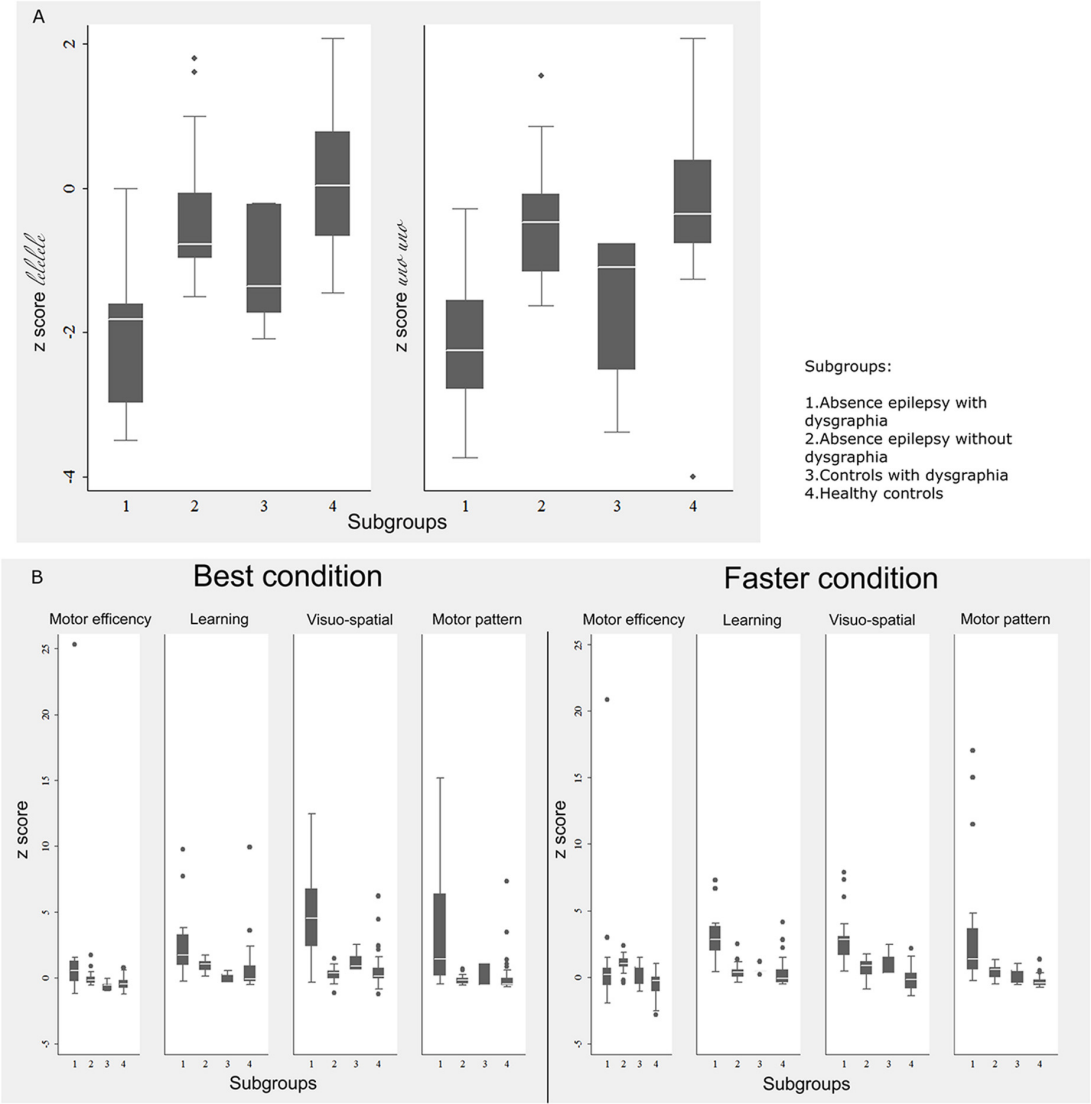
Handwriting fluency and DGM-P test box plot. Box plot of (**A**) handwriting fluency test and (**B**) DGM-P test with z-scores in the four subgroups.

Analysis of results of the “UNO” and “LE” tests in the four subgroups revealed the z-score distribution shown in the box plot (Fig 3A); children with absence epilepsy/dysgraphia performed more slowly than controls with dysgraphia, although a statistically significant difference was detected only for the “LE” test (t = 2.2247, P = 0.037).

Overall, children with absence epilepsy had worse handwriting quality than controls. Ordinal logistic regression showed that children with absence epilepsy as a whole, regardless of age, gender and class, performed worse than controls on both the ‘best’ and ‘faster’ conditions (RC best condition: -2.08 95% CI -2.98, -1.20; P<0.001; RC faster condition -2.93 95% CI -3.87, -1.98; P<0.001). The box plot of the four areas of interest of the DGM-P test with the z-score distribution within subgroups for ‘best’ and 'faster’ conditions is shown in Fig 3B. The *t*-test for unequal variances showed that patients with absence epilepsy/dysgraphia performed worse than controls with dysgraphia. A statistically significant difference was detected under the ‘best’ condition for learning (t = 3.859, P = 0.001) and visuo-spatial (t = 4.092, P<0.001), as well as under the ‘faster’ condition for learning (t = 5.521, P<0.001), visuo-spatial (t = 3.708, P = 0.001) and motor pattern (t = 2.569, P = 0.020).

Twelve patients with absence epilepsy/dysgraphia were re-tested 5 years after taking the first test. In eight of them we observed improved performances for both handwriting fluency and quality (75%). All twelve patients exhibited higher speed in the “UNO” test (t = -4.64, P<000.1) and obtained lower z-scores in the DGM-P test, consistent with better performances in the visuo-spatial component, both under ‘best’ (t = 4.215, P = 0.001) and ‘faster’ conditions (t = 7.653, P<0.001).

### Neurophysiology

The polymyographic study revealed normal EMG activity at rest and an abnormal pattern of muscle activation during handwriting (co-contraction of antagonist muscles, overflow of EMG activity to inappropriate muscles).

**Fig 4:**
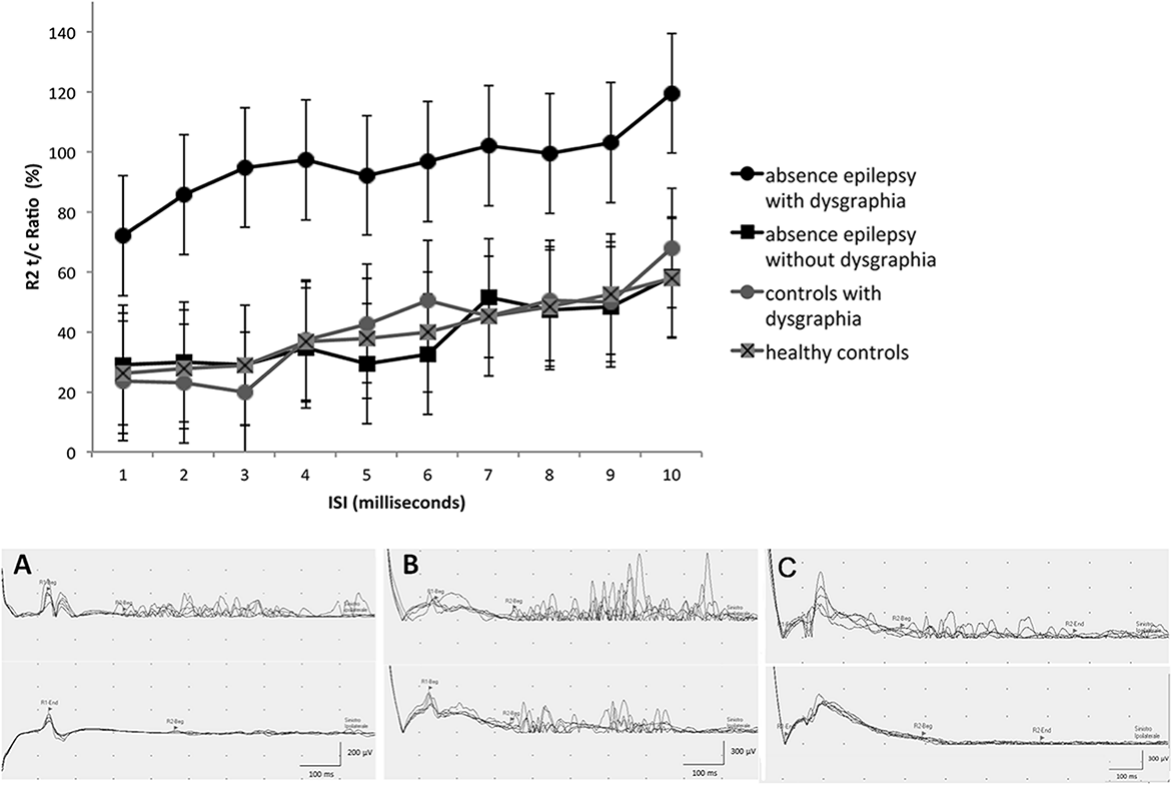
R2 blink reflex recovery cycle. Children with absence epilepsy/dysgraphia showed higher R2 values, compared to the three remaining groups. **(Lower part)** Blink reflex in a healthy control (A) and in a child with absence epilepsy/dysgraphia, showing no R2 suppression (B), which resolved at follow-up (C).

The R2 recovery cycle showed no suppression at short ISIs, with an initial R2 t/c ratio of 72% (ISI 100 milliseconds), progressively increasing up to 120% (ISI 1000 milliseconds). A statistically significant difference for each ISI value (P<0.001) was found when comparing the R2 recovery cycle of children with absence epilepsy/dysgraphia with responses obtained in children with absence epilepsy without dysgraphia treated with VPA, in controls with dysgraphia and in healthy controls (Fig 4, upper part). The five patients with absence epilepsy/dysgraphia in whom blink reflex and surface EMG recordings were repeated after 5 years exhibited a R2 component suppressed at short ISIs (F = 27.05, P = 0.0000), indicating a normalization of the blink reflex (Fig 4, lower part), and normal EMG traces during handwriting.

### Genetic testing

Direct sequencing and MLPA of the *SLC2A1* gene on genomic DNA revealed no alterations.

## Discussion

This study confirms our hypothesis, prompted by clinical observation, that the association between childhood absence epilepsy and dysgraphia is not coincidental, since it co-occurs at a significantly higher rate than in the control population (P = 0.016). It also suggests that dysgraphia, in the particular subpopulation of children with typical absence epilepsy, has a homogeneous, distinctive pathophysiological substrate. Both polymyography during handwriting and the R2 recovery curve of the blink reflex were consistent with findings observed in idiopathic dystonia [23–24]. Children with absence epilepsy without dysgraphia and controls with dysgraphia exhibited a normal blink reflex recovery cycle, confirming that increased excitability was observed only in children with the absence/dysgraphia association (P<0.001) and that valproate treatment did not influence the blink reflex response.

In addition, children with absence epilepsy/dysgraphia performed significantly worse than controls with dysgraphia in the “LE” test and in most handwriting quality tests, both under ‘best’ and ‘faster’ conditions. This finding provides further evidence for a different pathophysiology underlying dysgraphia in children with absence epilepsy with respect to mechanisms operating in the non-epilepsy subgroup. Clinical and EEG follow-up, re-testing of writing performances and repeated blink-reflex several years apart in children of the absence/dysgraphia cohort indicate that absences, dysgraphia and their accompanying neurophysiological abnormalities, are age-related manifestations with favorable outcome. Indeed, at follow up absence seizures were, as expected, no longer observed and the R2 recovery curve was suppressed, indicating that normalization of enhanced brainstem excitability had occurred. These changes were paralleled by an analogous improvement of the handwriting disorder, leading to resolution of dysgraphia.

Although dystonia is defined on clinical grounds [21], R2 abnormalities are frequently reported in various forms of dystonia. In children with absence epilepsy/dysgraphia neurophysiologic abnormalities further support, although they are not pathognomonic of, a dystonic mechanism involved in the pathogenesis. Increased R2 responses are usually observed in diffuse or cranial types of dystonia [24–25]. In children with absence epilepsy, the dysfunctional cortico-subcortical networks involved in absence seizures might suggest hyperexcitability also involving interneurons mediating the blink reflex, thus explaining the R2 abnormality even in this form of fine-tuned task induced focal disorder. Increased R2 responses were previously reported in patients with familial rolandic epilepsy and writer’s cramp [26].

Increased excitability of the R2 recovery curve of the blink reflex is thought to reflect defective suprasegmental control mechanisms [27].More specifically, the basal ganglia exert an inhibitory influence on bulbar interneurons and consequently modulate the blink reflex [27–28]. Loss of inhibitory processes in the nervous system is considered a predisposing substrate for dystonia [29].Reduced inhibitory output from the basal ganglia would result in increased excitability of brainstem interneuronal pathways mediating the R2 component of the response but could also engage the cerebral cortex via the thalamus [30].The basal ganglia and the thalamus have been repeatedly linked to the pathophysiology of absence seizures in experimental models [31].For instance, studies on GAERS rats suggest that the basal ganglia act as a remote control system for absence seizures, during which the cortico-subthalamo-pallidal network shows rhythmic bursting and striatal output neurons are silenced [32–33]. In children with absence seizures, EEG-triggered functional MRI demonstrates BOLD signal changes in the striato-thalamo-cortical network during 3 Hz generalized spike and wave discharges [34]. Abnormal water diffusion and volumetric abnormalities in the thalamus, caudate nucleus and putamen have been documented in patients with absence seizures [35]. Although available evidence is not sufficient to provide a conclusive, unitary theory on the pathophysiological substrate of absence epilepsy, it indicates that a dysfunctional cortical-basal ganglia network, or system, is at play in generating the absence phenomenology, at least in a number of patients.

It is therefore plausible that, in a subset of children with absence epilepsy, dysfunctional cortico-subcortical networks underlie the co-occurrence of absence seizures and of a mild form of dystonia, which is only manifested during handwriting. This ‘dystonic dysgraphia’ may be conceptually comparable to writer’s cramp in that it represents a peculiar form of focal, task-specific dystonia triggered in susceptible individuals by the overuse [30] caused by continuous repetitive finely-distributed muscle contractions required for handwriting.

Different genetic conditions are characterized by an association of epilepsy, with or without absence seizures, and dystonia [36]. An association of familial rolandic epilepsy with exercise-induced dyskinesia and dystonic writer’s cramp has been reported [26], but no causative gene has been found. Absence epilepsy and paroxysmal dyskinesia co-occur in some patients with *SLC2A1* mutations as part of the GLUT1 deficiency syndrome spectrum [22].

Although genetic factors are a major determinant of absence epilepsies [37], genetic causes are heterogeneous and complex and no gene has been clearly associated with the most common forms. Consequently, even the most clinically homogeneous syndromic subgroups, such as childhood absence epilepsy, are probably a discrete expression of a biological continuum [38]. Children with absence epilepsy and dysgraphia represent a subset of absence epilepsy manifesting subtle signs of dystonia, a clinical association that points to an underlying network dysfunction, rather than to a new syndrome.

The DSM V considers dysgraphia as an expression of a developmental coordination disorder of impaired written expression with emphasis on the motor component of written output skills (different from a specific learning disability in which grammatical or punctuation errors and poor paragraph organization prevail) [9]. Our findings contribute to understanding the heterogeneity of dysgraphia as a developmental coordination disorder by demonstrating that underlying dystonia is one cause of impaired written expression.

Our study has limitations. We found a dystonic background to be confined to children with dysgraphia and absence epilepsy but only explored a limited sample of children with dysgraphia without absence seizures. Indeed, after finding a normal blink reflex recovery cycle in all those studied we opted not to increase the sample further. We cannot exclude that a larger sample might have revealed an abnormal blink reflex response or signs of dystonia in some. We only performed polymyographic recordings in children with absence epilepsy/dysgraphia, but not in those with dysgraphia only as the clinical characteristics of the latter group did not suggest this relatively invasive investigation to be diagnostically yielding. Finally, we administered the initial handwriting screening battery only to a subgroup of patients with absence epilepsy whom we identified as “poor writers”. It is possible that in so doing we lost false negative children in the absence epilepsy group, thereby underestimating the prevalence of dysgraphia in our cohort with absence epilepsy.

## Conclusion

Our findings show that age related dysgraphia occurs at a significantly higher rate in children with absence epilepsy than in the control population. Clinical and neurophysiological findings, in this particular population, are consistent with an age-related dystonic origin of dysgraphia Considering that children spend 30% to 60% of their school day performing handwriting and other fine motor tasks [39], handwriting difficulties may seriously affect their daily life. Since our study was carried out over a 7-year period and included children enrolled at different ages, after observing a favorable outcome in older children with longer follow-up, we were able to provide families with reassurance that not only epilepsy but also dysgraphia would eventually disappear.

## Supporting Information

S1 File DGMP-test (Graph-motor and posture disorders of handwriting test). (DOC)

## References

[1] Leary LD, Wang D, Nordli DR, Engelstad K, De Vivo DC. Seizure characterization and electroencephalographic features in Glut-1 deficiency syndrome. Epilepsia 2003; 44: 701–707. 12752470 10.1046/j.1528-1157.2003.05302.x

[2] Du W, Bautista JF, Yang H, Diez-Sampedro A, You SA, Wang L et al Calcium-sensitive potassium channelopathy in human epilepsy and paroxysmal movement disorder. Nat Genet 2005; 37: 733–738. 15937479 10.1038/ng1585

[3] Wallace RH, Marini C, Petrou S, Harkin LA, Bowser DN, Panchal RG et al Mutant GABA(A) receptor gamma-2-subunit in childhood absence epilepsy and febrile seizures. Nat Genet 2001; 28: 49–52. 11326275 10.1038/ng0501-49

[4] Escayg A, De Waard M, Lee DD, Bichet D, Wolf P, Mayer T et al Coding and non coding variations of the human calcium-chanel β4 subunit gene *CACNB4* in patients with idiopathic generalized epilepsy and episodic ataxia. Am J Hum Genet 2000; 66: 1531–1539. 10762541 10.1086/302909 PMC1378014

[5] Jouvenceau A, Eunson LH, Spauschus A, Ramesh V, Zuberi SM, Kullmann DM et al Human epilepsy associated with dysfunction of the brain P/Q-type calcium channel. Lancet 2001; 358: 801–807. 11564488 10.1016/S0140-6736(01)05971-2

[6] Haug K, Warnstedt M, Alekov AK, Sander T, Ramìrez A, Poser B et al Mutations in *CLCN2* encoding a voltage-gated chloride channel are associated with idiopathic generalized epilepsies. Nat Genet 2003; 33: 527–532. 12612585 10.1038/ng1121

[7] Guerrini R, Sanchez-Carpintero R, Deonna T, Santucci M, Bhatia KP, Moreno T et al Early-onset absence epilepsy and paroxysmal dyskinesia. Epilepsia 2002; 43:1224–1229. 12366739 10.1046/j.1528-1157.2002.13802.x

[8] Deuel RK. Developmental dysgraphia and motor skills disorders. J Child Neurol 1995;10: S6–8. 7538525 10.1177/08830738950100S103

[9] American Psychiatric Association. Diagnostic and Statistical Manual of Mental Disorders, Fifth Edition Washington, DC: American Psychiatric Association; 2013.

[10] Karlsdottir R and Stefansson T. Problems in developing functional handwriting. Percept Mot Skills 2002; 94: 623–662. 12027360 10.2466/pms.2002.94.2.623

[11] Smith-Engelsman BCM, Niemeijer AS, Van Galen GP. Fine motor deficies in children diagnosed as DCD based on poor grapho-motor ability. Hum Mov Sci 2001;20: 161–182. 11471395 10.1016/s0167-9457(01)00033-1

[12] Riva D, Nichelli F, Devoti M. Developmental aspects of verbal fluency and confrontation naming in children. Brain Lang 2000; 71: 267–84. 10716861 10.1006/brln.1999.2166

[13] Sartori G, Job R, Tressoldi PE, editors. [Battery for the assessment of developmental dyslexia and dysorthographia]. Batteria per la valutazione della dislessia e della disortografia evolutiva Florence: Giunti OS; 1995.

[14] Tressoldi PE, Cornoldi C, editors [Battery for the assessment of writing skills of children from 7 to 13 years old]. Batteria per la valutazione della scrittura e della competenza ortografica nella scuola dell’obbligo Florence: Giunti OS; 2000.

[15] Bisiacchi P, Cendron M., Gugliotta M, Tressoldi PE, Vio C, editors. [Neuropsychological battery for children 5–11 years old]. BVN 5–11. Batteria di valutazione neuropsicologica per l’età evolutiva Trento: Erickson; 2005.

[16] Gugliotta M, Bisiacchi PS, Cendron M, Tressoldi PE, Vio C, editors. [Neuropsychological battery for adolescents 12–18 years old]. BVN 12–18. Batteria di valutazione neuropsicologica per l’adolescenza Trento: Erickson; 2009.

[17] Beery KE, Buktenica NA, Beery NA, editors. The Beery-Buktenica developmental test of visual-motor integration: administration, scoring and teaching manual Minneapolis: Pearson; 2004.

[18] Tressoldi PE, Cornoldi C, editors. [Battery for the assessment of writing skills of children from 7 to 13 years old]. Batteria per la valutazione della scrittura e della competenza ortografica nella scuola dell’obbligo Florence: Giunti OS; 2000.

[19] Borean M, Paciulli G, Bravar L, Zoia S, editors. [DGM-P: graph-motor and postural difficulties of handwriting test]. DGM-P: test per la valutazione delle difficoltà grafo-motorie e posturali della scrittura Trento: Erickson; 2012.

[20] Albanese A, Bhatia K, Bressman SB, Delong MR, Fahn S, Fung VS, et al Phenomenology and classification of dystonia: A consensus update. Mov Disord 2013; 28: 863–873. 10.1002/mds.25475 23649720 PMC3729880

[21] Sanger TD, Chen D, Fehlings DL, Hallett M, Lang AE, Mink JW, et al Definition and classification of hyperkinetic movements in childhood. Mov Disord 2010; 25: 1538–1549. 10.1002/mds.23088 20589866 PMC2929378

[22] Suls A, Mullen SA, Weber YG, Verhaert K, Ceulemans B, Guerrini R. Early-onset absence epilepsy caused by mutation in the glucose transporter GLUT1. Ann Neurol 2009; 66:415–419. 10.1002/ana.21724 19798636

[23] Berardelli A, Rothwell JC, Day BL, Marsden CD. Pathophysiology of blepharospasm and oromandibular dystonia. Brain 1985;108: 593–608. 4041776 10.1093/brain/108.3.593

[24] Tolosa E, Montserrat L, Bayes A. Blink reflex studies in focal dystonias: enhanced excitability of brainstem interneurons in cranial dystonia and spasmodic torticollis. Mov Disord 1988; 3: 61–69. 3173365 10.1002/mds.870030108

[25] Pauletti G, Berardelli A, Cruccu G, Agostino R, Manfredi M. Blink reflex and the masseter inhibitory reflex in patients with dystonia. Mov Disord. 1993; 8: 495–500. 8232360 10.1002/mds.870080414

[26] Guerrini R, Bonanni P, Nardocci N, Parmeggiani L, Piccirilli M, De Fusco M et al Autosomal recessive rolandic epilepsy with paroxysmal exercise-induced dystonia and writer's cramp: delineation of the syndrome and gene mapping to chromosome 16p12-11.2. Ann Neurol 1999; 45: 344–352. 10072049 10.1002/1531-8249(199903)45:3<344::aid-ana10>3.0.co;2-9

[27] Esteban A. A neurophysiological approach to brainstem reflexes. Blink reflex. Neurophysiol Clin 1999; 29: 7–38. 10093816 10.1016/S0987-7053(99)80039-2

[28] Aramideh M, Eekhof JL, Bour LJ, Koelman JH, Speelman JD, Ongerboer de Visser BW. Electromyography and recovery of the blink reflex in involuntary eyelid closure: a comparative study. Journal of Neurology, Neurosurgery and Psychiatry 1995; 58: 692–698. 7608667 10.1136/jnnp.58.6.692 PMC1073546

[29] Quartarone A, Hallett M. Emerging concepts in the physiological basis of dystonia. Mov Disord 2013; 28: 958–967. 10.1002/mds.25532 23893452 PMC4159671

[30] Berardelli A, Rothwell JC, Hallett M, Thompson PD, Manfredi M, Marsden CD. The pathophysiology of primary dystonia. Brain 1998;121:1195–1212. 9679773 10.1093/brain/121.7.1195

[31] Deransart C, Vercueil L, Marescaux C, Depaulis A. The role of basal ganglia in the control of generalized absence seizures. Epilepsy Res 1998; 32: 213–223. 9761322 10.1016/s0920-1211(98)00053-9

[32] Slaght SJ, Paz T, Chavez M, Deniau JM, Mahon S, Charpier S. On the activity of the corticostriatal networks during spike and wave discharged in a genetic model of absence epilepsy. J Neurosci 2004; 28: 6816–6825. 10.1523/JNEUROSCI.1449-04.2004 PMC6729718 15282287

[33] Paz T, Deniau JM, Charpier S. Rhythmic bursting in the cortico-subthalamo-pallidal network during spontaneous genetically determined spike and wave discharged. J Neurosci 2005; 25: 2092–2101. 15728849 10.1523/JNEUROSCI.4689-04.2005 PMC6726056

[34] Moeller F, Siebner HR, Wolff S, Muhle H, Granert O, Jansen O et al Simultaneous EEG-fMRI in drug-naive children with newly diagnosed absence epilepsy. Epilepsia 2008; 49:1510–1519. 10.1111/j.1528-1167.2008.01626.x 18435752

[35] Luo C, Xia Y, Li Q, Xue K, Lai Y, Gong Q et al Diffusion and volumetry abnormalities in subcortical nuclei of patients with absence seizures. Epilepsia 2011; 52: 1092–1099. 10.1111/j.1528-1167.2011.03045.x 21453358

[36] Guerrini R. Idiopathic epilepsy and paroxysmal dyskinesia. Epilepsia 2001;42 (Suppl 3): S36–41. 10.1046/j.1528-1157.2001.042suppl.3036.x 11520321

[37] Berg AT, Berkovic SF, Brodie MJ, Buchhalter J, Cross JH, van Emde Boas W et al Revised terminology and concepts for organization of seizures and epilepsies: report of the ILAE Commission on Classification and Terminology, 2005–2009. Epilepsia 2010;51:676–685. 10.1111/j.1528-1167.2010.02522.x 20196795

[38] Berkovic SF, Andermann F, Andermann E, Gloor P. Concepts of absence epilepsies: discrete syndromes or biological continuum? Neurology 1987;37:993–1000. 3108696 10.1212/wnl.37.6.993

[39] McHale K, Cermak SA. Fine motor activities in elementary school:preliminary findings and provisional implications for children with fine motor problems. Am J Occup Ther 1992; 46: 898–903. 1463061 10.5014/ajot.46.10.898

