# Supplementary material for: Dysgraphia as a Mild Expression of Dystonia in Children with Absence Epilepsy
Source: PLoS One. 2015 Jul 1;10(7):e0130883. doi: 10.1371/journal.pone.0130883 (PMC4488862; doi:10.1371/journal.pone.0130883)
Supplement: S1 File — (DOC) [file pone.0130883.s001.doc]

**DGMP-test (*Graph-motor and posture disorders of handwriting test*)**

List of 12 indexes providing information about handwriting speed, learning and quality.

**Handwriting speed**

1. **Handwriting speed:** we counted number of letters completed, including letter corrections and time required for transcription (number of letters/sec).

**Handwriting learning**

1. **Error learning:** we counted number of letters in which we observed error on movement and directions of letter formation (for example: acute turns in connecting letters and letters performed with no functional movement).

**Handwriting quality**

1. **Self-corrections:** we counted number of letters in which we observed self-corrections.
2. **Floating letters:** we counted number of letters written above or below 1.5 mm lines of paper.
3. **Dysmetria:** we counted number of letters in which we observed irregularities in joining letters.
4. **Confusion between similar letters:** we counted number of letters replaced by similar ones.
5. **Ascending/descending strokes reduced in size:** we counted number of letters in which ascending/descending strokes were reduced in size.
6. **Unrecognizable letters:** we counted number of unrecognizable letters.
7. **Collision of letters**: we counted number of decreased space between letters.
8. **Maximum amplitude of the fluctuation**: we evaluated the maximum length of ascending and descending strokes from the lines of paper.
9. **Incorrect relative letter height (medium size letters):** we evaluated the height differences between the smallest and the largest letters of medium size.
10. **Incorrect relative letter height (letters with ascending/descending strokes):** we evaluated the height differences between the smallest and the largest letters with ascending/descending strokes.

**Four areas of interest in handwriting :** the test also provided information about 4 areas of interest: visuo-spatial component, motor efficiency, motor pattern, learning, identified by principal component analysis ^1^

| **Indexes** | **Areas of interest in handwriting** |
| --- | --- |
| 1. **Handwriting speed**   **9. Collision of letters**  **7. Ascending/descending strokes reduced in size** | **Motor efficiency** |
| **4. Floating letters**  **10. Maximum amplitude of the fluctuation**  **11. Incorrect relative letter height (medium sized eeeeeeeletters)**  **12. Incorrect relative letter height (letters with ascending/descending strokes)**    **5. Dysmetria** | **Visuo-spatial component** |
| **3. Self-corrections**  **6. Confusion between similar letters**  **8. Unrecognizable letters** | **Motor pattern** |
| **2. Error learning** | **Learning** |

**Reference**

1. Borean M, Paciulli G, Bravar L, Zoia S, editors. [DGM-P: graph-motor and postural difficulties of handwriting test]. DGM-P: test per la valutazione delle difficoltà grafo-motorie e posturali della scrittura Trento: Erickson; 2012. Italian.
